# Liquiritin exhibits anti-acute lung injury activities through suppressing the JNK/Nur77/c-Jun pathway

**DOI:** 10.1186/s13020-023-00739-3

**Published:** 2023-04-03

**Authors:** Hongling Zhou, Tangjia Yang, Zibin Lu, Xuemei He, Jingyu Quan, Shanhong Liu, Yuyao Chen, Kangtai Wu, Huihui Cao, Junshan Liu, Linzhong Yu

**Affiliations:** 1 Third Level Research Laboratory of State Administration of Traditional Chinese Medicine, School of Traditional Chinese Medicine, Southern Medical University, Guangzhou, 510515 People’s Republic of China; 2 Guangdong Provincial Key Laboratory of Chinese Medicine Pharmaceutics, School of Traditional Chinese Medicine, Southern Medical University, Guangzhou, 510515 People’s Republic of China; 3 Department of Pharmacy, Zhujiang Hospital, Southern Medical University, Guangzhou, 510282 People’s Republic of China

**Keywords:** Liquiritin, Lipopolysaccharide, Acute lung injury, JNK, Nur77, c-Jun

## Abstract

**Background:**

Licorice (*Glycyrrhiza uralensis* Fisch.), a well-known traditional medicine, is traditionally used for the treatment of respiratory disorders, such as cough, sore throat, asthma and bronchitis. We aim to investigate the effects of liquiritin (LQ), the main bioactive compound in licorice against acute lung injury (ALI) and explore the potential mechanism.

**Methods:**

Lipopolysaccharide (LPS) was used to induce inflammation in RAW264.7 cells and zebrafish. Intratracheal instillation of 3 mg/kg of LPS was used for induction an ALI mice model. The concentrations of IL-6 and TNF-α were tested using the enzyme linked immunosorbent assay. Western blot analysis was used to detect the expression of JNK/Nur77/c-Jun related proteins. Protein levels in bronchoalveolar lavage fluid (BALF) was measured by BCA protein assay. The effect of JNK on Nur77 transcriptional activity was determined by luciferase reporter assay, while electrophoretic mobility shift assay was used to examine the c-Jun DNA binding activity.

**Results:**

LQ has significant anti-inflammatory effects in zebrafish and RAW264.7 cells. LQ inhibited the expression levels of p-JNK (Thr183/Tyr185), p-Nur77 (Ser351) and p-c-Jun (Ser63), while elevated the Nur77 expression level. Inhibition of JNK by a specific inhibitor or small interfering RNA enhanced the regulatory effect of LQ on Nur77/c-Jun, while JNK agonist abrogated LQ-mediated effects. Moreover, Nur77-luciferase reporter activity was suppressed after JNK overexpression. The effects of LQ on the expression level of c-Jun and the binding activity of c-Jun with DNA were attenuated after Nur77 siRNA treatment. LQ significantly ameliorated LPS-induced ALI with the reduction of lung water content and BALF protein content, the downregulation of TNF-α and IL-6 levels in lung BALF and the suppression of JNK/Nur77/c-Jun signaling, which can be reversed by a specific JNK agonist.

**Conclusion:**

Our results indicated that LQ exerts significant protective effects against LPS-induced inflammation both in vivo and in vitro via suppressing the activation of JNK, and consequently inhibiting the Nur77/c-Jun signaling pathway. Our study suggests that LQ may be a potential therapeutic candidate for ALI and inflammatory disorders.

## Background

Acute lung injury (ALI) is a primary cause of death in many severe infectious respiratory diseases, such as sepsis, pneumonia and COVID-19 [1]. ALI is characterized by capillary leakage, inflammatory cells infiltration, arterial hypoxemia, and pulmonary edema [2]. Currently, the existing ALI treatment methods mainly includes supportive treatment and drug intervention treatment. The lung protection strategy of mechanical ventilation is considered as the only supportive treatment to improve the survival [3]. Moreover, drug interventions include nutrients, antioxidants, protease inhibitors, complement inhibitors and so on [4]. Despite a large of interventions, effective therapies to apparently improve patient’s survival and quality of life remain elusive [5]. Therefore, searching for effective anti-ALI medications has substantial implications for clinical practice.

Bacterial sepsis is the most common risk factor of ALI. LPS can induce sepsis syndrome accompanied by key features of ALI, and thereby is commonly used for establishing ALI model in rodents [6]. After LPS stimulation, mitogen-activated protein kinases (MAPKs), including extracellular signal-regulated kinases (ERKs), c-Jun N-terminal kinases (JNKs), and p38 kinases are activated [7]. Studies showed that activation of JNK and p38 aggravated sepsis induced organ damage, whereas the mortality rate of septic mice was significantly reduced after inhibition of JNK and p38 [8]. Consistently, inhibition of ERK efficiently attenuates LPS-induced pulmonary inflammation in murine ALI models [9].

Activated JNKs can activate downstream transcriptional factors c-Jun and Nur77 [10]. JNK was reported phosphorylate c-Jun on residues Ser63 and Ser73, which lead to inhibition of c-Jun ubiquitination and degradation, thereby causes an accumulation of the c-Jun [11]. It was reported that the deletion of c-Jun in CD11c^+^ dendritic cells can reduce psoriasis-like skin inflammation induced by the TLR7 agonist Imiquimod [12]. Moreover, rosmarinic acid ameliorated the signs of hepatic inflammation in extrahepatic cholestasis rats model through the c-Jun/AP-1 signaling cascade [13]. The scarcity of Nur77 induced macrophages to differentiate into M1 pro-inflammatory phenotype, while the activation of Nur77 attenuated LPS-induced ALI by inhibiting endothelin-1. Taken together, these finding suggested that JNK/Nur77/c-Jun signaling plays a critical role in inflammation-related diseases. However, whether and how JNK plays a role in Nur77-mediated biological effects in inflammation remains unknown.

**Fig. 1:**
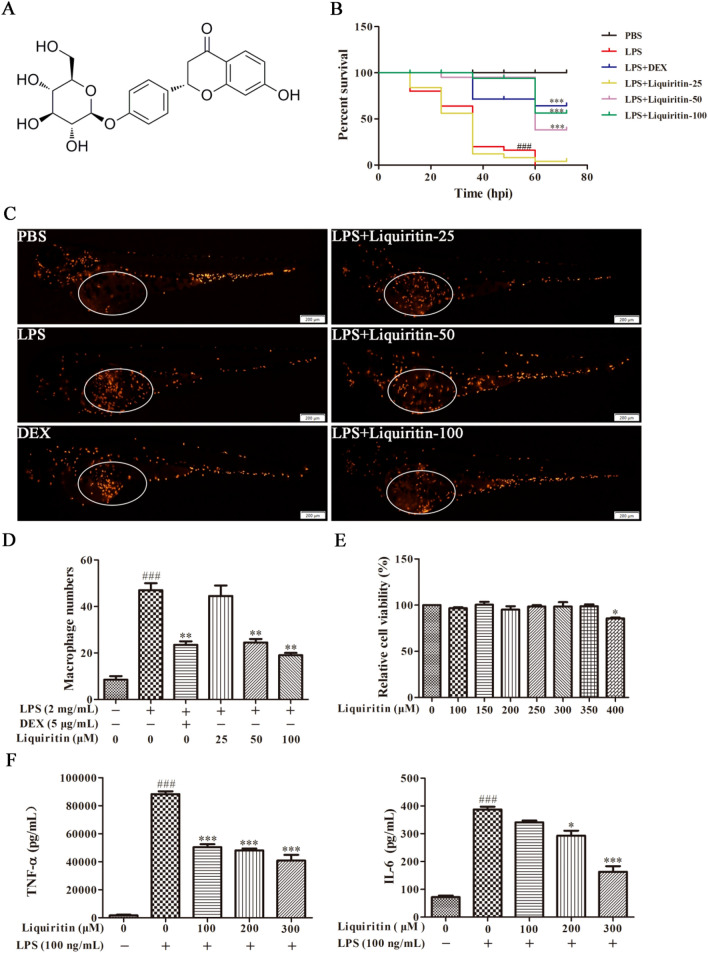
LQ has significant anti-inflammatory effects in vitro and in vivo. **A** The chemical structure of LQ. **B** LQ improved the survival rate of LPS-microinjected zebrafish larvae. One hundred and eighty larvae were randomly divided into 6 groups (n = 30). larvae were exposed to indicated dosages of LQ after PBS or LPS (2 mg/mL) yolks microinjection, then the mortalities of the larvae were observed for 72 h. **C** Representative fluorescence images and (**D**) statistical analysis of macrophages in yolk were shown. **E** RAW264.7 cells viability was determined by MTT assay. **F** RAW264.7 macrophage cells were pretreated with LQ (100, 200, 300 µM) for 2 h, then LPS (100 ng/mL) were added for another 22 h incubation. The supernatants were collected and the concentrations of TNF-α and IL-6 were determined by ELISA. Data were presented as mean ± SEM, *n* = 3, ^###^*P* < 0.001 versus control; *P < 0.05 versus LPS, **P < 0.01 versus LPS, ***P < 0.001 versus LPS. One-way ANOVA, post hoc comparisons, Turkey’s test

Traditional Chinese medicine licorice (*Glycyrrhiza uralensis* Fisch.) is commonly used for the treatment of respiratory disorders including cough, sore throat, asthma and bronchitis [14]. Licorice tablets and licorice oral solution are widely used as antitussives and expectorants in clinical practice [15]. Modern pharmacological research shows that licorice has diverse actions, such as anti-inflammation, anti-virus, anti-bacterial, immune regulation, etc. [16]. Liquiritin (LQ, Fig. 1A) is one of the characteristic flavonoid components in licorice and is also considered as one of the index components of licorice. Research demonstrated that LQ exhibits anti-inflammatory effects on various inflammation-related diseases, such as skin injury, hepatic inflammatory injury and rheumatoid arthritis [17]. Moreover, five active components of licorice extract, Glycyrol, Isolicoflavonol, Licochalcone A, 18β-glycyrrhetinic acid, and Licoisoflavone A, can alleviate paraquat-induced ALI in mice by upregulating CYP_450_ and Nrf_2_ pathways [18]. Meanwhile, Schaftoside, the active ingredient of licorice, can treat COVID-19 by inhibiting 3CL^pro^ and PL^pro^ of SARS-CoV-2 virus and modulating immune response and inflammation of host cells [19]. Furthermore, Glycyrrhizin was shown to alleviate acute lung injury by inhibiting NLRP3 inflammasome [20]. However, the role of LQ and its underlying mechanisms in ALI remain to be further investigated. Therefore, in the present study, we investigated the anti-ALI effects and underlying mechanisms of LQ both in vitro and in vivo.

## Materials and methods

### Cell culture

Mouse macrophages RAW264.7 and human embryonic kidney cell line HEK293T were obtained from Cell Bank of the Chinese Academy of Science (Shanghai, China). Both cells were maintained in DMEM with 10% (*v*/*v*) fetal bovine serum (Gibco) and 1% (*v*/*v*) penicillin/streptomycin (Gibco). Then cells were cultured in an incubator at 37 ^o^C.

### Cell viability assay

RAW264.7 cells were seeded in a 96-well plate (8.0 × 10^3^ cells *per* well) and then incubated with indicated concentrations of LQ (100, 150, 200, 250, 300, 350, 400 µM, Herbpurify, Chengdu, China, CAS: 551-15-5) for 24 h. Subsequently, 30 µL of MTT working solution was added to each well. After 4 h, the supernatant was replaced with 100 µl of DMSO solution. The absorbance was measured at 490 nm using a microplate spectrophotometer (Thermo Fisher Scientific, USA).

### Enzyme-linked immunosorbent assay

RAW264.7 cells were seeded in a 24-well plate (1 × 10^5^ cells *per* well). After 24 h incubation, DMEM containing different concentrations of LQ was added to cells for 2 h, and then LPS was added for co-treatment 22 h. Supernatants were collected for detection of IL-6 and TNF-α concentrations by ELISA kits (Dakewei, Beijing, China).

### Western blotting

The collected cells were lysed with RIPA containing protease and phosphatase inhibitors and protein concentrations were determined by the BCA assay kit (Thermo Fisher Scientific). Then proteins were resolved in 10% SDS-PAGE and further transferred to PVDF membranes (Millipore, USA). Subsequently, membranes were probed with indicated primary and secondary antibodies. Finally, the bands were detected using the Fluor Chem E™ system (Protein Simple, San Francisco, USA).

### Small interfering RNA transient transfection

RAW264.7 cells were transfected with 120 nM of JNK siRNA and Negative siRNA using lipofectamine^®^ 2000 (Invitrogen, Grand Island, USA) according to manufacturer’s introduction. 24 h post-transfection, cells were treated with LQ (300 µM) for 2 h and then LPS was added for co-treatment 22 h. Then, cells and supernatants were collected for immunoblotting and ELISA, respectively.

### Dual-Luciferase reporter assay

HEK293T cells were plated in a 24-well plate (5 × 10^4^ cells *per* well). Cells were co-transfected with the reporter plasmid pGL3-Luc and control plasmid pRL-TK with or without MAPK8 overexpressing plasmid pcDNA3.1 (GenePharm, China) using Lipofectamine® 2000 (Invitrogen). 12 h later, LQ (300 µM) were added for another 12 h. Then cells were lysed using lysis buffer for 30 min at RT and the luciferase Renilla activity was determined by Dual-Luciferase Reporter Assay System (Promega, USA) following the manufacturer^’^s instructions.

### Electrophoretic mobility shift assay (EMSA)

Nuclear proteins from RAW264.7 macrophages were incubated with reaction buffer with or without Nur77 biotin-labeled probe. The mixture was separated on a 6.5% nondenaturing PAGE and transferred to nylon membrane. DNA-protein crosslinking was then conducted by using a UVP crosslinker (Analytik Jena, Jena, Germany). Blocking buffer containing horseradish peroxidase-conjugated streptavidin was incubated with the membrane, followed by imaged by FluorChem E™ system. The double-stranded oligonucleotides probes to detect Nur77 binding was 5’-GAG TTT TAA AAG GTC ATG CTC AAT- 3’ (GenePharma, Suzhou, China).

### Zebrafish embryo and larvae maintenance

The transgenic line Tg (*mpeg1:dsRed*) expressing fluorescence on macrophage was kindly provided by South China University of Technology (Guangzhou, China). Zebrafish were raised in a circulating tank system with light for 14 h and darkness for 10 h at 28℃ (water conductivity: 500 to 550 µS/cm; pH of 6.9 to 7.2). The embryos were collected by mating male and female zebrafish in a 1:2 ratio.

### LPS-yolk microinjection model and drug administration

3 days post-fertilization (dpf) zebrafish were anesthetized by adding 0.02% tricaine and fixed on the agarose plate. Then 2 nL of LPS (2 mg/mL) was injected into the yolk sac of zebrafish to create local inflammation model, while the negative control group was injected with the equal amount of PBS. The zebrafish were transferred to 6-well plates containing egg water, followed by administration with LQ (25, 50, 100 µM) or dexamethasone (DEX, 5 µg/mL). Finally, the images of fluorescent macrophages were recorded by a microscope (MVX10, Olympus, Japan).

### Animals

5–6 weeks old male BALB/c mice (18–22 g) were obtained from Guangdong Medical Laboratory Animal Center (Guangzhou, China). The mice were reared under specific pathogen-free conditions at temperatures of 23–25 ℃, relative humidity of 40–70%, and 12-h dark/light cycles for 1 week to acclimate them prior to the experiment. Animal research was conducted in strict accordance with the Guide for the Care and Use of Laboratory Animals and approved by the Committee for the experimental use of animals at Southern Medical University (No. L2019108).

### Grouping and modeling

Forty-eight mice were stochastically divided into six groups (n = 8 *per* group).

Control group: animals were intragastric administrated with 200 µL of saline daily for consecutive 7 days. At day 7, animals were intratracheal instillation with saline.

LPS group: animals were intragastric administrated with 200 µL of saline for 7 days. Then, 3 mg/kg LPS was injected into the trachea of mice.

LQ-40, LQ-80, and DEX group: mice were intraperitoneal injection with 40 or 80 mg/kg of LQ, or 5 mg/kg of dexamethasone daily for consecutive 7 days, respectively. At the last day, 3 mg/kg LPS was injected into the trachea of mice.

Anisomycin (a JNK agonist) group: mice were intraperitoneal injection with 15 mg/kg of anisomycin, and 1 h later, they were treated with 80 mg/kg of LQ. After consecutive 7 days treatment, 3 mg/kg LPS was injected into the trachea of mice.

After intratracheal instillation with LPS for 8 h, mice were anesthetized by intraperitoneal injection of 1.5% pentobarbital. Lung tissues and bronchoalveolar lavage fluid (BALF) were taken for further analysis.

### Histopathological observation of lung tissues

Part of the lung tissue was fixed with 4% formaldehyde, embedded with paraffin, and then stained with hematoxylin and eosin (H&E). The pathological changes of lung tissue were observed by an IX 53 light microscope (Olympus, Tokyo, Japan) [21].

### Lung wet/dry weight (W/D) ration measurement

The harvested lung tissues were weighed to record the wet weight (W). The lung tissues were then placed in a 60 ^o^C incubator for 72 h to complete dehydration. The dry lung tissues were weighed again, and the dry weight (D) was recorded. The water content of lung tissue was calculated by determining the W/D ratio [21].

### Protein levels in BALF

Once mice were sacrificed, trachea were separated. Lungs were lavaged with 0.5 mL of PBS each time, and repeated twice. The BALF was then centrifuged and supernatant was collected for detection of protein content using a BCA protein assay kit.

### Statistical analysis

Standard statistical test analysis was conducted using GraphPad Prism 8, and the data were expressed as mean ± SEM. One-way analysis of variance (ANOVA) was used to compare among multiple groups. The *P* less than 0.05 was considered statistically significant.

## Results

### LQ exerts significant anti-inflammatory effects in vivo and in vitro

Zebrafish is a widely accepted and used animal model in infectious diseases for its unique advantages in genetic manipulation and real-time imaging [22]. As shown in Fig. 1B, zebrafish mortality reached 100% at 60 h after LPS microinjection, while LQ significantly improved the survival rate of LPS-infected larvae, with up to 60% at 100 µM. This result indicated that LQ could protect zebrafish from lethal LPS challenge.

The *mpeg1:DsRed* transgenic zebrafish can intuitively observe the behavior of macrophages during the inflammatory process. Therefore, we further evaluated the macrophage migration after LQ treatment in LPS-injected larvae. As shown in Fig. 1C, D, macrophages considerably recruit to the yolk after LPS stimulation, while treatment with LQ dose-dependently reduced aggregation of macrophages to the LPS-injected site.

To evaluate the anti-inflammatory effect of LQ in vitro, we firstly investigated the nontoxic concentration of LQ on RAW264.7 cells. Results showed that treatment with 100 ~ 350 µM LQ for 24 h did not significantly influence cell viability (Fig. 1E). Therefore, concentrations of LQ below 350 µM were chosen in the following study. Next, we explored the effect of LQ on LPS-stimulated pro-inflammatory cytokines. As displayed in Fig. 1F, LPS remarkably stimulated the release of TNF-α and IL-6 and these phenomena were inhibited after treatment with LQ (100, 200, 300 µM) in a dose-dependent manner.

### LQ suppresses the activation of JNK in LPS-stimulated RAW264.7 macrophage cells

**Fig. 2:**
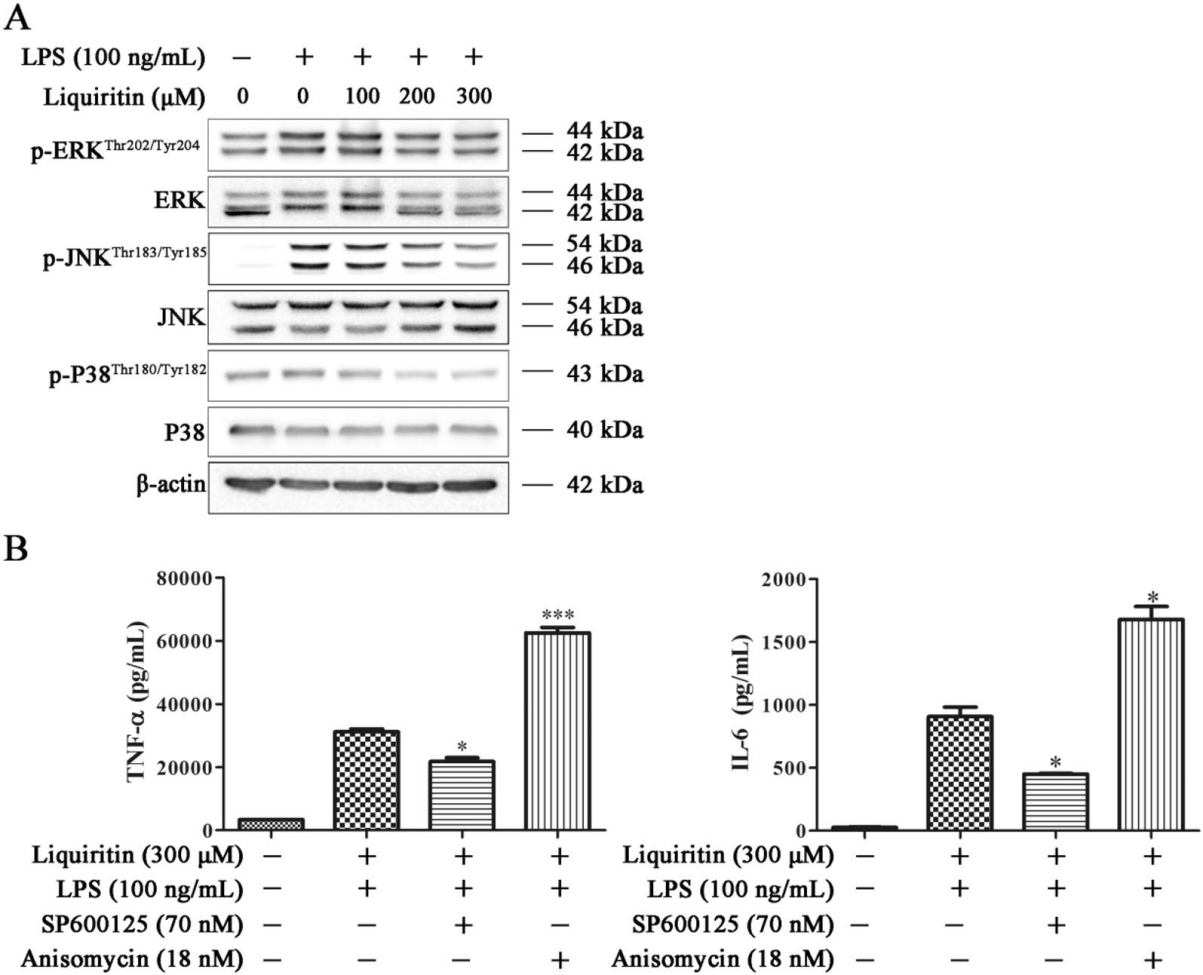
LQ exerts anti-inflammatory effects through inhibiting JNK. **A** LQ inhibited the phosphorylation of JNK. RAW264.7 macrophage cells were treated with LQ (100–300 µM) for 2 h, then LPS (100 ng/mL) were treated for another 22 h. Protein expression levels of p-JNK, JNK, p-ERK, ERK, p-p38 and p38 were determined by Western blotting. **B** LQ inhibits the pro-inflammatory cytokines by suppressing of JNK. RAW264.7 cells were treated with JNK inhibitor SP600125 (70 nM) or JNK activator anisomycin (18 nM) for 2 h before the administration of LQ, and treated with LPS for another 22 h. Cell supernatants were collected, and the concentration of TNF-α and IL-6 were measured by ELISA. Data are presented as mean ± SEM, *n* = 3, ^*^P < 0.05, ^***^P < 0.001 *versus* LQ group. One-way ANOVA, *post hoc* comparisons, Turkey’s test. Columns, means; error bars, SEM

Accumulating data showed that MAPK activation is critical in regulating LPS-induced inflammation response. MAPK comprise a family of protein-serine/threonine kinase, including ERKs, JNKs and p38s. To study whether the MAPK signaling pathway was involved in the anti-inflammatory effects of LQ, expression and phosphorylation levels of ERK, JNK and p38 were detected by Western blotting. Results showed that LPS increased the expression levels of p-ERK^Thr202/Tyr204^, p-JNK^Thr183/Tyr185^ and p-p38^Thr180/Tyr182^. LQ treatment significantly inhibited JNK phosphorylation in a dose-dependent manner, while had no obvious influence on p-ERK and p-p38 (Fig. 2A).

To examine whether JNK involved in the anti-inflammatory effects of LQ, we used a JNK chemical inhibitor (SP600125) and a JNK activator (anisomycin) to inhibit or activate JNK, then determined effects of LQ on TNF-α and IL-6. As shown in Fig. 2B, in the presence of SP600125, the inhibitory effect of LQ on TNF-α and IL-6 were enhanced, while anisomycin reduced LQ-mediated inhibitory effects. The above data together indicated that JNK is involved in the anti-inflammatory effect of LQ.

### LQ decreases Nur77 and c-Jun by suppressing JNK

**Fig. 3:**
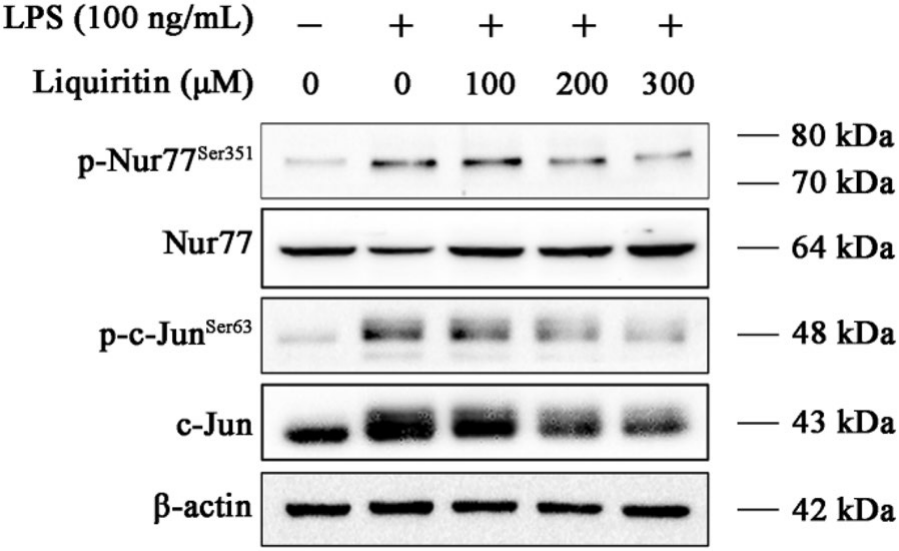
LQ supresses the activation of Nur77 and c-Jun in LPS-stimulated RAW264.7 macrophage cells. RAW264.7 cells were treated with indicated concentrations of LQ for 2 h, then they were stimulated with LPS (100 ng/mL) for another 22 h. The expressions of p-Nur77^Ser351^, Nur77, p-c-Jun^Ser63^, and c-Jun were determined using Western blotting

JNKs are involved in cell proliferation, survival, death and differentiation through activating downstream targets including c-Jun and Nur77 [10]. We then examined the effects of LQ on c-Jun and Nur77 in LPS-stimulated RAW264.7 macrophage cells. As shown in Fig. 3, the expression levels of c-Jun, p-c-Jun ^Ser63^ and p-Nur77^Ser351^ were elevated after LPS stimulation, while the expression level of Nur77 was decreased, which suggested that LQ inhibited Nur77 and c-Jun activation.

**Fig. 4:**
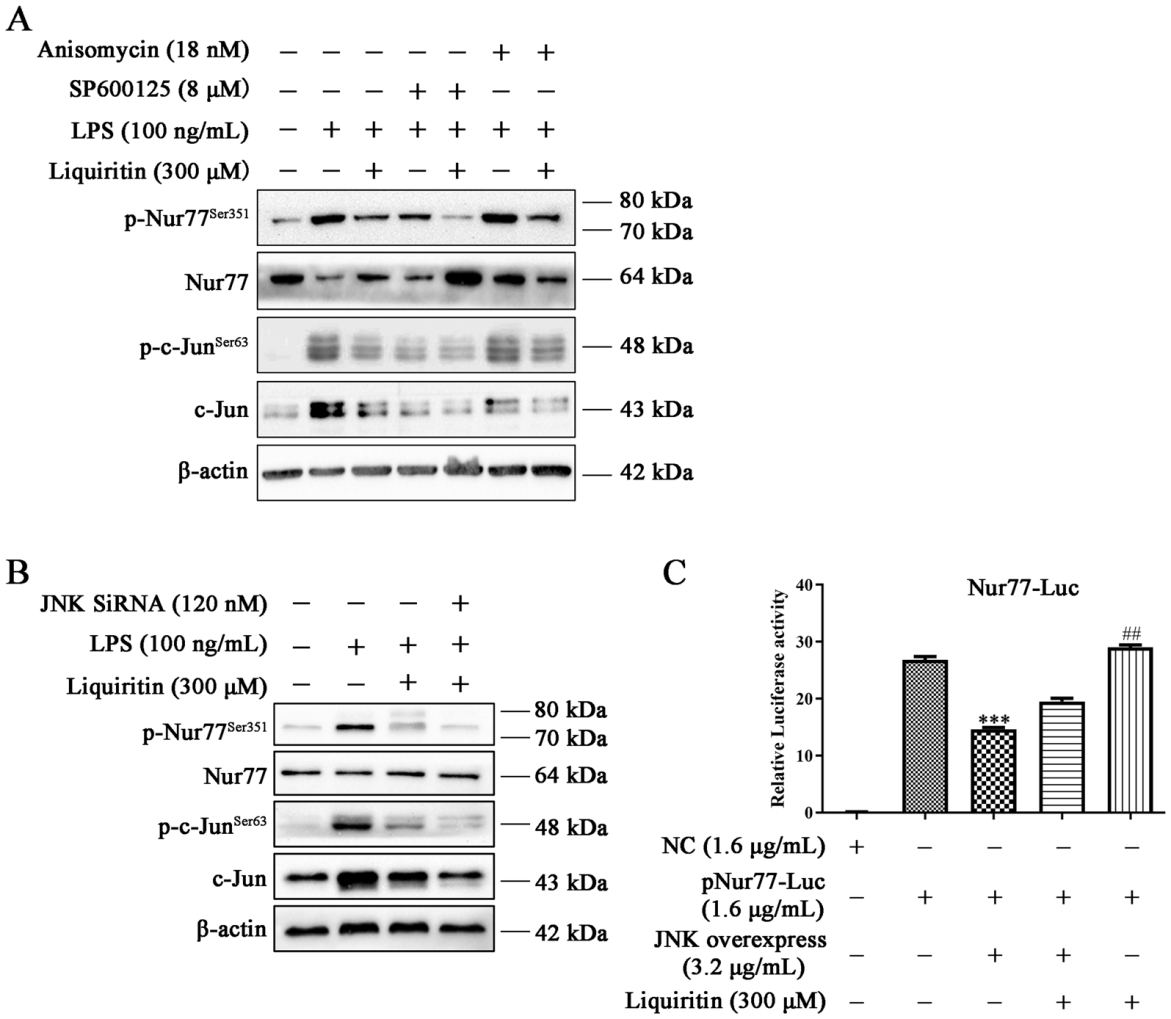
LQ inhibits Nur77/c-Jun by JNK suppression. **A** RAW264.7 cells were pretreated with LQ, SP600125 (8 µM) or Anisomycin (18 nM) for 2 h and then incubated with LPS (100 ng/mL) for another 22 h. Western blotting was employed to detect the expression levels of p-Nur77^Ser351^, Nur77, p-c-Jun, and c-Jun^Ser63^. **B** JNK is involved in LQ-induced inhibition of the Nur77/c-Jun pathway. RAW264.7 cells were transfected with JNK siRNA for 24 h, and then treated with LQ 2 h, followed by LPS stimulation for another 22 h. The protein levels of p-Nur77 (Ser351), Nur77, p-c-Jun (Ser63), c-Jun were determined. **C** JNK overexpression inhibited the luciferase activity of Nur77. HEK293 T cells were co-transfected with JNK overexpression plasmid, Nur77-Luc and TK-Luc plasmids for 24 h. Then, cells were incubated with LQ (300 µM) for 24 h. The luciferase activity of Nur77 was determined using a Dual-Glo luciferase assay system kit. Data are presented as mean ± SEM, ^***^P < 0.001 *versus* Nur77-luc group, ^##^*P* < 0.01 *versus* JNK overexpress group. One-way ANOVA, *post hoc* comparisons, Turkey’s test. Columns, means; error bars, SEM

We further investigated whether JNK regulated LQ-mediated Nur77 and c-Jun inactivation. As shown in Fig. 4, the inhibitory effect of LQ on p-Nur77^Ser351^, p-c-Jun^Ser63^ and c-Jun were retarded by JNK activator anisomycin (Fig. 4A). While inhibiting JNK by a specific inhibitor SP600125 (Fig. 4A) or siRNA transfection (Fig. 4B) enhanced the effects of LQ on the expression levels of p-Nur77^Ser351^, p-c-Jun^Ser63^ and c-Jun.

We also determined the effect of JNK on Nur77 transcriptional activity by luciferase reporter assay. As shown in Fig. 4C, LQ increased the transcriptional activity of Nur77, while JNK over-expression significantly suppressed the effect of LQ. These results demonstrated that LQ inhibited Nur77 and c-Jun activation through JNK inhibition.

### LQ inhibits c-Jun by the upregulation of Nur77

**Fig. 5:**
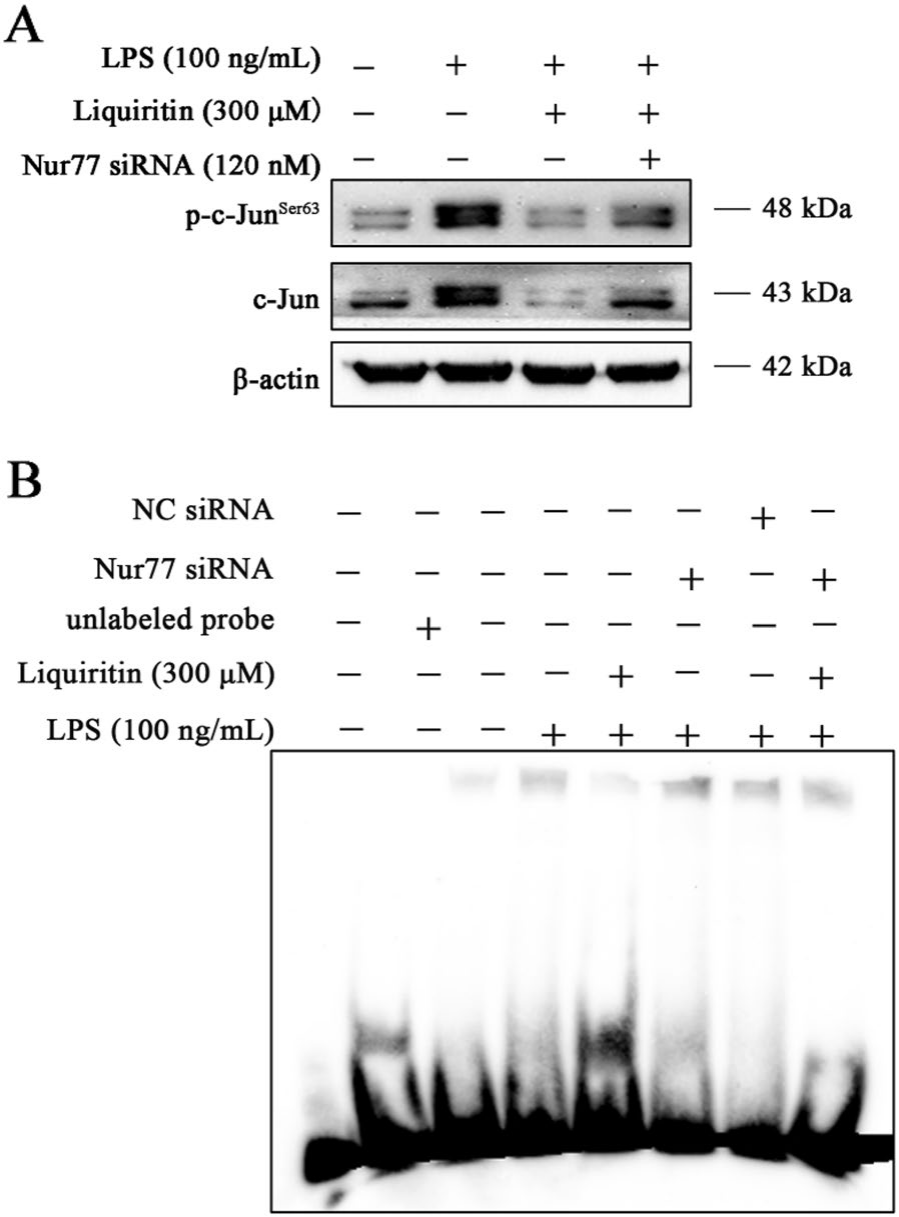
LQ decreases c-Jun expression level and DNA binding activity by the up-regulation of Nur77. RAW264.7 cells were transfected with Nur77 siRNA for 24 h, after that, cells were treated with LQ for 2 h and then stimulated with LPS for another 22 h. The total protein and nuclear proteins were extracted. **A** Expression levels of p-c-Jun (Ser63) and c-Jun were determined by Western blotting. **B** The c-Jun DNA binding activity was examined by EMSA

To determine the relationship between Nur77 and c-Jun, cells were transfected with Nur77 siRNA. Data showed that blockage of Nur77 attenuated the inhibitory effect of LQ on p-c-Jun^Ser63^ and c-Jun (Fig. 5A). Moreover, EMSA result showed that Nur77 inhibition could up-regulate the DNA binding activity of c-Jun in LPS-stimulated RAW264.7 cells (Fig. 5B). These results demonstrated that LQ reduced c-Jun by increasing Nur77.

### LQ attenuates LPS-induced acute lung injury (ALI) by inhibiting JNK in murine ALI mode

**Fig. 6:**
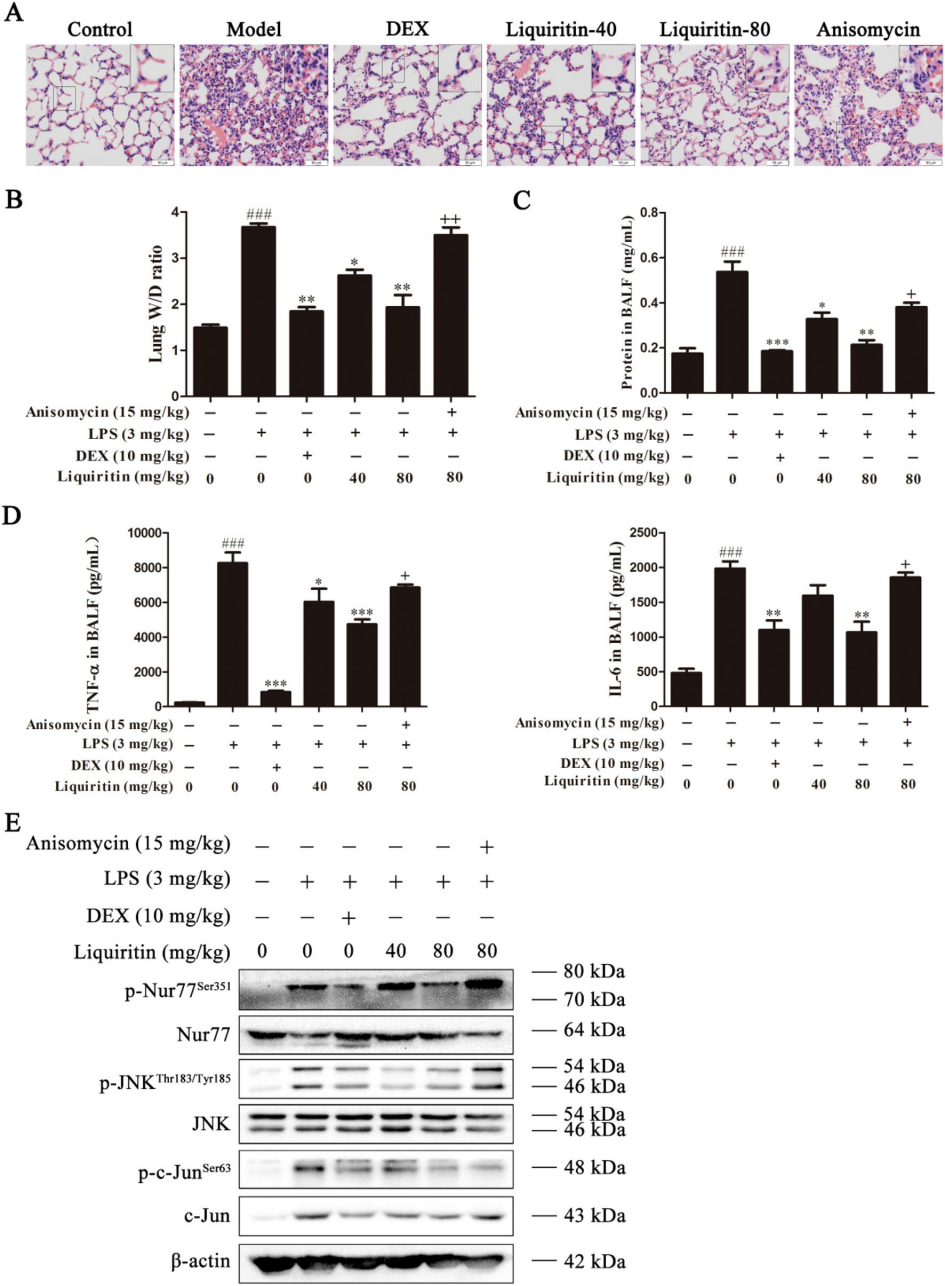
LQ inhibits the activation of the JNK/Nur77/c-Jun pathway in LPS-induced ALI in mice. **A** Lung tissues were dehydrated, embedded, sliced, H&E stained and photographed (40 ×). (**B** − **E**) Anisomycin counteracts the decrease of the inflammation and edema induced by LQ in ALI mice. At the end of experiment, lungs were separated and weighed to get the W/D ratio (**B**). BALF was obtained to detect the total protein concentration by the BCA protein assay kit (**C**). The levels of TNF-α and IL-6 (**D**) were evaluated by ELISA. **E** The lung tissues were homogenized and lysis to obtain total protein. The expression levels of Nur77, p-Nur77 (Ser351), JNK, p-JNK (Thr183/Tyr185), c-Jun, p-c-Jun (Ser63) in total cell lysates were determined by Western blotting. Data were shown as mean ± SEM. ^###^*P* < 0.001 *versus* control group, ^*^P < 0.05, ^**^P < 0.01, ^***^P < 0.001 *versus* LPS group; ^+^*P* < 0.05, ^++^*P* < 0.01 *versus* LQ-80 group. one-way ANOVA, *post hoc* comparisons, Turkey’s test

LPS is widely used to establish murine ALI model, which is characterized by lung edema, hypoxemia, thickening of the alveolar wall and neutrophil infiltration. Therefore, we determined whether LQ could prevent LPS-induced ALI through inhibiting JNK. Histological analyses revealed that LQ dose-dependently reduced ALI mice edema, hyperaemia, alveolar wall thicken and suppressed inflammatory cell infiltration, while the effects of LQ can be counteracted by a JNK activator anisomycin (Fig. 6A). Moreover, LQ also down-regulated edematous and inflammation indexes, including lung W/D ration (Fig. 6B), total protein (Fig. 6C) and the levels of TNF-α and IL-6 (Fig. 6D) in BALF of ALI mice. Moreover, 80 mg/kg of LQ exhibited similar anti-ALI effects compared with DEX. These results indicated that LQ possesses the significant anti-ALI activity, which may partially attribute to its inhibitory effects on JNK.

We further detected the expression levels of Nur77, JNK and c-Jun in the lung tissues of ALI mice. As shown in Fig. 6E, LQ suppressed the phosphorylation of Nur77 at Ser351, phosphorylation of JNK at Thr183/Tyr185 and phosphorylation of c-Jun at Ser63 and up-regulated the expression level of Nur77. Critically, the effects of LQ could be abolished by anisomycin, confirmed the important role of JNK in LQ-mediated effects. Collectively, these data indicated that LQ alleviated ALI through JNK inhibition.

## Discussion

ALI is a severe pulmonary disease that usually accompanied with overwhelmed inflammatory responses. The production of excess inflammatory mediators and cytokines can mediate, amplify, and perpetuate the lung injury process, which in turn promote ALI pathogenesis and development [23]. Despite several complement therapies and conventional pharmacological treatments are available, the mortality rate of ALI remains high [5]. Therefore, further studies to find effective therapies and drugs for ALI are urgently needed. In this study, we provide in vivo and in vitro evidences that LQ ameliorates LPS-induced ALI through the inhibition of JNK/Nur77/c-Jun pathway. These results demonstrated that LQ could be considered as a potential agent for the treatment of ALI.

In recent years, zebrafish (*Danio rerio*) have emerged as an ideal model for disease studies and drug screening due to the advantages such as rapid life cycle, low cost and transparency [24]. Zebrafish present a fully innate immune system with macrophages and neutrophils for host defense and zebrafish infection models have been established for numerous pathogens [25]. Our previous study showed that microinjection of LPS into the zebrafish yolk, CuSO_4_ and tail fin injury can be used to induce the zebrafish acute inflammation [26]. In this study, we established zebrafish yolk LPS-microinjected inflammatory model on Tg (*mpeg1:DsRed*) zebrafish larvae to observe macrophages aggregation. Our data showed that LQ protected against LPS-induced zebrafish death *via* the suppression of macrophage recruitment.

The MAPK pathway plays a vital role in inflammation and immune responses. Three members of MAPKs-p38, ERK, and JNK are activated in LPS-induced sepsis and ALI [27]. Studies have shown that p38MAPK specific inhibitor SB203580 and JNK selective inhibitor SP600125 can reduce the inflammatory cytokine release in BALF, improve the pulmonary histology, and reduce the mortality rate of LPS-induced ALI [8, 28]. Our data also indicated that p38, ERK, and JNK were phosphorylated after LPS stimulation, while only JNK was markedly inhibited by LQ. Additionally, SP600125 and LQ played a synergistic effect in reducing the expression of TNF-α and IL-6. In the contrast, JNK activator anisomycin significantly aggravated the inflammatory response, which counteracted the inhibitory effect of LQ in vivo and *in vitro.* These findings demonstrated that LQ attenuated inflammation *via* the inhibition of JNK.

Many in vivo experiments demonstrated that Nur77 possesses anti-inflammatory effects [29]. Nur77 deficiency exacerbates a variety of inflammation-driven pathologies, such as LPS-induced sepsis, acute liver inflammation and atherosclerosis [30], while the overexpression of Nur77 can suppress the inflammatory status of macrophages. Our data showed that LQ decreased the phosphorylation of Nur77 and increased the expression of Nur77. Moreover, considerable evidence indicated that JNK plays important roles in Nur77 activation. JNK can effectively phosphorylate Nur77 mainly at the N-terminus, thereby inhibiting the DNA binding and transactivation activities of Nur77 [31]. Considering LQ also inhibited JNK, we examined whether LQ regulated Nur77 expression and promoter activity by inhibiting the expression of JNK. Our results showed the JNK agonist anisomycin could counteract the effects of LQ on the expression and phosphorylation of Nur77, while the JNK inhibitor SP600125 could enhance the effects, suggesting that LQ suppresses the phosphorylation of Nur77 by inhibiting JNK activation.

JNK activated by extracellular stimulation can phosphorylate c-Jun transcription factor, thereby regulating the transcription and expression of downstream genes [11]. Previous studies suggested that blocking the JNK/C-Jun pathway could inhibit LPS-induced production of pro-inflammatory factors in RAW264.7 cells, thereby displaying an anti-inflammatory potentials [32]. In the present study, we found that LQ can significantly down-regulated the expression of c-Jun and p-c-Jun at Ser63, thus inhibiting the release of pro-inflammatory factors TNF-α and IL-6. Meanwhile, the collective administration of SP600125 and LQ displayed better inhibitory activities on the protein levels of c-Jun and p-c-Jun at Ser63 when compared to LQ treatment. Additionally, Anisomycin could antagonize the inhibition of LQ on these two proteins both in vitro and in vivo. Our results suggested that LQ exerts anti-inflammatory effects through inhibiting the expression of JNK and further inhibiting the c-Jun and p-c-Jun.

Recently, evidence suggests that Nur77 suppresses the expression of c-Jun, which in turn inhibits c-Jun promoter activity [33]. Indeed, we found that the inhibitory effect of LQ on c-Jun activity was significantly weakened when blockage of Nur77, indicating that c-Jun may be the downstream of Nur77. Previous research also demonstrated that c-Jun inhibited the transcriptional activation and DNA binding activity of Nur77 through direct interaction with Nur77 [34], which suggested Nur77 and c-Jun exist a mutual regulatory relationship. Further research is needed to clarify the regulatory relationship of LQ on Nur77 and c-Jun.

Our research demonstrated that LQ exerts anti-ALI activities, which may be related to the anti-inflammatory and anti-oxidant effects of flavonoids. For example, Gan An He Ji oral liquid, which contains licorice liquid extracts and paregoric, has proven to be clinically effective in the treatment of pneumonia, and its active ingredients (Liquiritin, Liquiritigenin, Glycyrrhizin, Glycyrrhetinic acid, Daidzin, and Formononetin) have been shown to be effective in alleviating pneumonia and lung injury by downregulating iNOS in cells and mice [35]. In addition, LQ was reported could inhibit the elevation of NF-κB, TRPV1 and TRPA1 protein expression in LPS-induced ALI mice [36]. Our data further confirmed that LQ display significant anti-ALI effects by downregulating the JNK pathway. Collectively, LQ exhibits great potentials in the treatment of lung inflammation and deserves more in-depth study.

## Conclusion

**Fig. 7:**
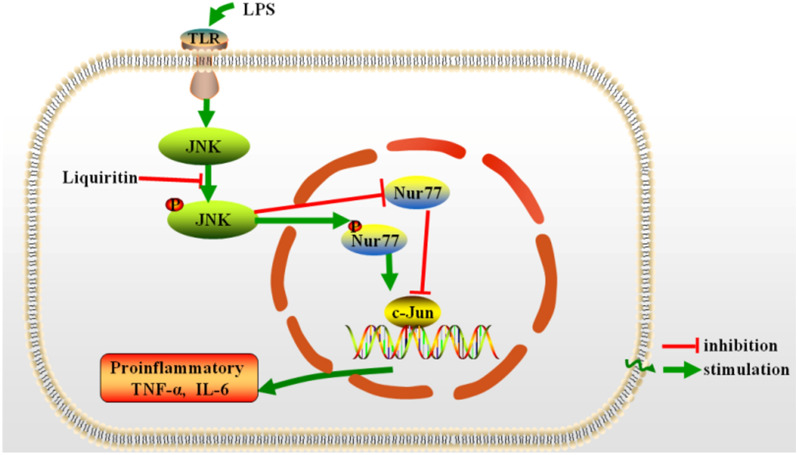
The schematic of the anti-inflammatory mechanisms of LQ in LPS-induced ALI. LQ blocks the activation of JNK and inhibits the phosphorylation of Nur77, which suppressed the DNA binding activity of c-Jun and the release of pro-inflammatory cytokines TNF-α and IL-6

In summary, our results firstly demonstrated that LQ exerts significant protective effects against LPS-induced inflammation both in vivo and in vitro via suppressing the activation of JNK, and consequently inhibiting the Nur77/c-Jun signaling pathway. These results clarify the anti-ALI rationale of LQ and suggest that LQ could be a valuable therapeutic candidate in the treatment of ALI (Fig. 7)

## Data Availability

The data used to support the findings of this study are available from the corresponding author upon request.

## Abbreviations

ALI: Acute lung injury
AP-1: Activator protein-1
ARDS: Acute respiratory distress syndrome
BALF: Bronchoalveolar lavage fluid
DEX: Dexamethasone
dpf: Days post fertilization
ECL: Enhanced chamiluminescence
ELISA: Enzyme-linked immunosorbent assay
EMSA: Electrophoretic mobility shift assay
ERK: Extracellular regulated protein kinases
H&E: Hematoxylin and eosin
hpi: Hours post injection
IL-6: Interleukin-6
JNKs: The c-Jun N-terminal kinases
LPS: Lipopolysaccharide
MAPKs: Mitogen-activated protein kinases
MTT: 3-[4, 5-dimethylthiazol-2-yl]-2, 5-diphenyltetrazoliumbromide
PBS: Phosphate-buffered saline
RT: Room temperature
siRNA: Small interfering RNA
TNF-α: Tumor necrosis factor-alpha
